# Content-based microarray search using differential expression profiles

**DOI:** 10.1186/1471-2105-11-603

**Published:** 2010-12-21

**Authors:** Jesse M Engreitz, Alexander A Morgan, Joel T Dudley, Rong Chen, Rahul Thathoo, Russ B Altman, Atul J Butte

**Affiliations:** 1 Department of Bioengineering, Stanford University School of Medicine, Stanford, CA, USA; 2 Biomedical Informatics Training Program, Stanford University School of Medicine, Stanford, CA, USA; 3 Department of Pediatrics and Department of Medicine, Stanford University School of Medicine, Stanford, CA, USA; 4 Department of Computer Science, Stanford University, Stanford, CA, USA; 5 Departments of Genetics and Medicine, Stanford University School of Medicine, Stanford, CA, USA; 6 Lucile Packard Children's Hospital, Stanford University, Stanford, CA, USA

## Abstract

**Background:**

With the expansion of public repositories such as the Gene Expression Omnibus (GEO), we are rapidly cataloging cellular transcriptional responses to diverse experimental conditions. Methods that query these repositories based on gene expression content, rather than textual annotations, may enable more effective experiment retrieval as well as the discovery of novel associations between drugs, diseases, and other perturbations.

**Results:**

We develop methods to retrieve gene expression experiments that differentially express the same transcriptional programs as a query experiment. Avoiding thresholds, we generate differential expression profiles that include a score for each gene measured in an experiment. We use existing and novel dimension reduction and correlation measures to rank relevant experiments in an entirely data-driven manner, allowing emergent features of the data to drive the results. A combination of matrix decomposition and *p*-weighted Pearson correlation proves the most suitable for comparing differential expression profiles. We apply this method to index all GEO DataSets, and demonstrate the utility of our approach by identifying pathways and conditions relevant to transcription factors Nanog and FoxO3.

**Conclusions:**

Content-based gene expression search generates relevant hypotheses for biological inquiry. Experiments across platforms, tissue types, and protocols inform the analysis of new datasets.

## Background

With the development of the DNA microarray and other technologies that probe gene expression on an "omic" scale, we are now able to discover associations between biological conditions based on their molecular underpinnings. Seminal work by Golub et al. [1] classified leukemia samples by their global gene expression profiles, demonstrating that transcriptomic signatures can aid in functional prediction and improve our molecular understanding of disease. Hughes et al. [2] predicted the effects of novel gene deletions and chemical treatments by profiling yeast mutants and comparing new arrays to this reference. More recent studies examined cellular transcriptional response to drug treatment [3,4] and disease [5,6] in order to identify novel relationships between apparently unrelated conditions and compounds. This work not only demonstrated the utility of expression-based discovery, but also suggested that functional studies about drugs and diseases can utilize data from different platforms and cell types. This general approach to hypothesis generation - namely, finding associations between diverse conditions based on gene expression - has great potential to further biological and biomedical research if implemented on a large scale.

Here we develop methods for content-based gene expression search using an entire experiment as a query. That is, given an input experiment comparing case to control, we aim to identify other experiments that show similar patterns of differential expression. This concept is exemplified by the Connectivity Map [3], which searches for relationships between treatment-control comparisons for small molecules. While the Connectivity Map focused on drug treatment and disease, a similar approach across a sufficiently large data source would allow for the identification of associations between gene knockdowns, diseases, drugs, and myriad other perturbations and phenotypes. Public repositories provide a wealth of data amenable to this task. The largest of these repositories, the National Center for Biotechnology Information (NCBI) Gene Expression Omnibus (GEO) [7], now contains over 400,000 individual samples from more than 17,000 experiments detailing the molecular characteristics of diverse cell types, diseases, and drug treatments. The European Bioinformatics Institute (EBI) ArrayExpress Repository [8] and Stanford Microarray Database [9] host additional data. While GEO supports searches of its content based on free-text and controlled-vocabulary annotations, there is increasing interest in methods for querying microarray databases based on the molecular measurements themselves [10-14]. The power of this approach would grow with the size of the repository.

Current methods for content-based search typically involve a two-step process: they identify a gene set of interest and then search for experiments in which this gene set is important. Several groups have introduced methods for identifying experiments that co-express [15] or differentially express [11] a given gene set. Recently, EBI implemented the Gene Expression Atlas, which provides this latter functionality over their curated array archive [13]. These methods, however, require that both the query and target experiments differentially express genes above some hard threshold, and thus may miss more subtle or noisy relationships [16]. Other approaches, typified by Gene Set Enrichment Analysis (GSEA) [16], partially bypass this requirement by comparing a subset of genes to ranked profiles, using a hard threshold for the query experiment and a soft threshold for the queried experiments [3,4].

While previous approaches require designating a group of differentially expressed genes, we explore the possibility of using as a query a differential expression (DE) profile, consisting of a complete list of features and associated expression scores. By examining all genes shared between query and queried experiments, we aim to identify experimental conditions and perturbations that exhibit similar transcriptional responses. A successful strategy in this effort should reconcile differences between species, platform types, and normalization methods, as well as overcome the confounding effects of noise and technical replicability. To achieve this, we consider combinations of methods for three tasks: data representation, dimension reduction, and search algorithm.

First, we consider the problem of data representation. Typical microarray analysis methods represent differential expression as a fold-change, comparing the expression in one set of samples to that in another [17]. However, because public expression databases consist of a broad range of data types and experimental modalities, rank-based representations are often employed to account for the disparities in the distributions of observed data [4,10]. Here we compare both parametric and nonparametric data representations to determine the best approach for comparing DE profiles. We also consider an alternate representation of gene expression data, and construct DE profiles based on the *p*-value of differential expression.

A second challenge is that gene expression profiles from high-throughput technologies consist of up to tens of thousands of measurements per sample. In addition to the computational complexity involved in handling these large datasets, high dimensionality often confounds data mining techniques [16,18]. In particular, high-dimensional, multimodal data lends itself to over-fitting and reduced performance [19]. Many solutions to this problem have been proposed, of which dimensionality reduction is the foremost. Matrix decomposition [20,21], feature selection [22], and module or gene-set based approaches [16,23] attempt to capture the most relevant data while removing redundant or noisy features [18].

Given an appropriate data representation for differential expression, the final challenge is how best to calculate the similarity between two experiments. While Fujibuchi et al. use Spearman rank correlation to compare individual microarrays [10], it is not clear whether a similar approach is appropriate for DE profiles. Several recent studies use a modified Pearson correlation measure on rank-normalized profiles [4,5,24]. Other work suggests that weighting expression values by each gene's variance may improve classification and analysis [25,26].

**Figure 1:**
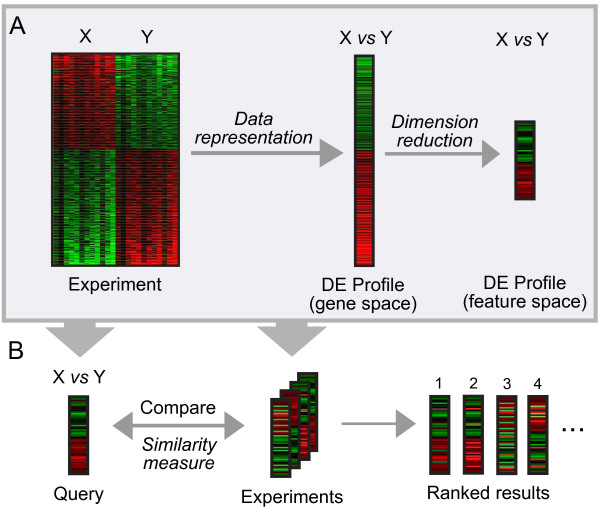
**Schematic diagram of our approach**. (A) Creation of differential expression (DE) profiles. An experiment comparing condition X with condition Y is condensed to a single DE profile in gene space. Dimension reduction is applied to create a DE profile in a reduced feature space. (B) Searching a library of DE profiles. The query profile is compared to the DE profiles of all other experiments in our disease compendium (or in GEO) using a similarity measure. Results are ranked by their similarity to the query profile. Italics indicate variable steps in our pipeline.

To begin to address these challenges, we test several search schema representing combinations of data representation, dimension reduction, and correlation measures in a curated collection of 32 disease-related GEO experiments. We create DE profiles to represent the changes in transcription between normal and disease samples (Figure 1A), and evaluate the performance of our schema in retrieving experiments that measure the same disease as a query experiment (Figure 1B). We find that a projection method for dimension reduction performs as well or better than search in gene-space, and introduce an intuitive *p*-value weighted correlation coefficient that performs the best in our test compendium. Using the most successful parameters, we exhaustively index GEO DataSets (GDS) totaling 31,453 arrays and 2,089 experiments. We demonstrate the utility of our method by querying our database of DE profiles with several experiments examining transcription factor knockdown in embryonic and neural stem cells. This work demonstrates the feasibility of content-based microarray search for the large-scale discovery of functional links between gene expression experiments.

## Results

### Evaluation of data representation and similarity measures

**Figure 2:**
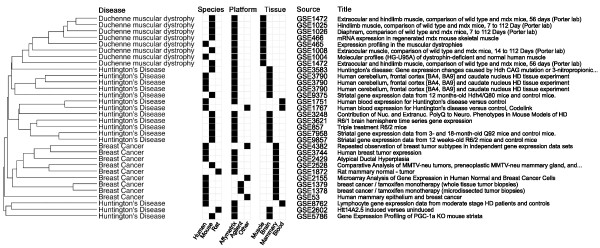
**Disease compendium**. Our collection of 32 disease-related experiments represents several combinations of species, platforms, and tissues. Differential expression profiles based on log fold-change were generated for each experiment, then mapped to human genes through Homologene. We compared DE profiles using Pearson correlation and applied hierarchical clustering to find that the profiles cluster primarily by disease and tissue. One GEO Series appears more than once: GSE3790 provides three profiles that cluster together, comparing normal to diseased tissue in cerebellum, frontal cortex, and caudate nucleus.

To develop a differential-expression search utility for GEO, we first evaluated various data processing pipelines in a compendium of 32 microarray experiments comparing normal to diseased tissue. This collection included three diseases with differing genetic origin: Duchenne muscular dystrophy, Huntington's disease, and breast cancer. The studies originated from different laboratories and measured primary human disease samples as well as animal disease models. Although these experiments represented various combinations of species, platform, and normalization techniques, they clustered primarily by disease and tissue (Figure 2). To search this collection of experiments based on differential expression (DE), we created a DE profile for each experiment (32 total), consisting of a list of features (e.g., genes) each with an associated score (e.g., fold-change). We permuted various processing and ranking techniques to search for the combination of parameters that was best able to identify other experiments of the same disease given a query experiment. We evaluated the sensitivity and specificity of these processing pipelines with leave-one-out cross-validation: we used each experiment to query the remaining 31 experiments with the goal of identifying other experiments that measure the same disease.

**Figure 3:**
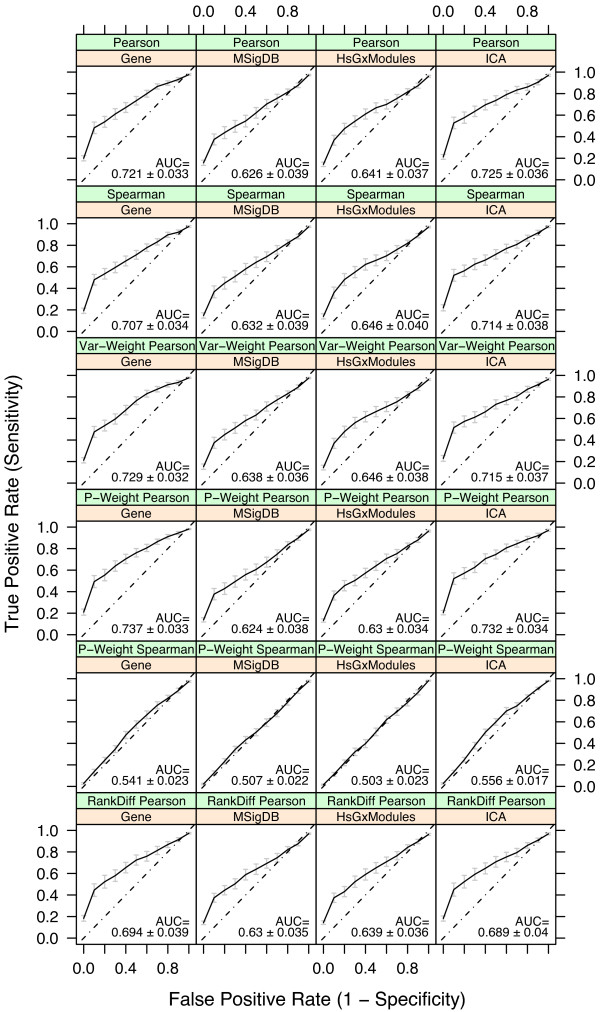
**Evaluation of dimension reduction methods and similarity measures**. Comparison of four dimension reduction methods and six similarity measures using leave-one-out cross-validation in our disease compendium. Bars and AUC estimates indicate standard errors for curves averaged over all cross-validation trials. The three similarity measures based on Pearson correlation outperform the rank-based approaches, with the *p*-weighted Pearson correlation proving the best at identifying other experiments of the same disease. The ICA projection method for dimension reduction outperforms the module-based approaches, and performs comparably to gene-level analysis. HsGxModules = Human Gene Expression Modules (see Methods).

First we compared the effects of data representation on our ability to retrieve relevant experiments. Using both Pearson and Spearman correlation, we found that representing differential expression as a log fold-change nominally outperformed rank- and *p*-value-based representations (see Additional file 1). For subsequent tests we focused on the fold-change representation. Next we evaluated all combinations of four dimension reduction methods and six similarity measures (Figure 3). For dimension reduction, projection onto features identified by independent component analysis (ICA, see Methods) [27] outperformed module-based representations. While none of the dimension reduction methods made convincing improvements over the gene-level analysis, the ICA projection method did not result in a loss of information, successfully recapitulating performance in gene-space using a significantly reduced number of features. For similarity measures, unweighted and *p*-value weighted Pearson correlations nominally outperformed Spearman correlation for analysis in gene- and ICA feature-space, resulting in the highest overall areas under the receiver operating curve. *P*-weighted Spearman correlation performed the worst for all dimension reduction methods.

The receiver operating curves for the best-performing search methods indicated that, on average, about 50% of the true positives could be recovered with greater than 90% specificity. This high specificity is important for search because typically the first few results, rather than a complete list, are examined. To evaluate the performance of our search over the top results in each search, we calculated the "precision at 4" for each of the 32 experiments, permuting labels to create a null model (see Additional file 2). The average precision for Duchenne muscular dystrophy and Huntington's disease exceeded the random model at a 95% confidence interval for 13/15 and 8/8 experiments, respectively. The "precision at 4" for breast cancer, a genetically complex disease, was also high, but it significantly surpassed the random model in only 4/9 experiments.

### Constructing a network of GEO differential expression experiments

**Figure 4:**
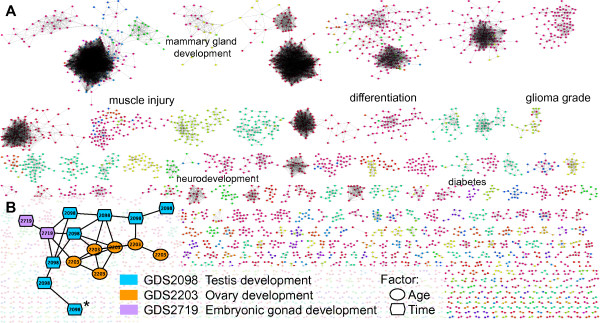
**Network of GEO differential expression profiles**. (A) We calculated *p*-weighted correlations between 9,415 differential expression profiles from GEO and connected highly similar profiles (*q *< 0.001). Nodes are colored according to experimental variable (e.g., time). Dense clusters tend to represent multiple profiles from the same experiment. We identified multi-experiment clusters corresponding to processes including muscle injury, mammary gland development, and glioma grade. For a high resolution figure, see Additional files 3 and 4. (B) Close-up of a multi-experiment cluster. DE profile nodes are re-colored to correspond to the GEO DataSet from which they originate, and node shape represents experimental variables. *Compares gestation day 14 to gestation day 16.

Our comparisons of data processing methods and similarity measures suggested that the *p*-weighted Pearson correlation in gene- or ICA-space is most effective at retrieving biologically relevant DE profiles. Because the ICA-based method reduced the number of features by a factor of 50, we used this approach to systematically index GEO DataSets. We created a total of 9,415 DE profiles, one for each combination of NCBI-curated experimental conditions within a dataset. For example, if a dataset had "1 hr", "2 hr", and "4 hr" groups, we generated a comparison for each of "1 hr vs 2 hr", "2 hr vs 4 hr", and "1 hr vs 4 hr." We excluded 364 comparisons that failed to successfully map to human genes through Homologene; these experiments measured primarily bacterial and plant species. To visualize this set of profiles, we calculated pairwise similarities using *p*-weighted Pearson correlation and created a network of differential expression experiments (Figure 4A, Additional files 3, 4). Random comparisons were used to build a null distribution of similarity scores. With a strict cutoff (*q *< 0.001), highly-connected subnetworks consisting of multiple profiles from the same dataset emerged. Clusters of profiles from multiple experiments also were apparent, linking datasets that examined related biological processes and perturbations. Figure 4B shows a multi-experiment cluster examining gonad development in mouse, consisting of differential expression profiles from GDS2098, GDS2203, and GDS2719. Each profile compares gonad tissue at two developmental stages, between 10 and 18 days post coitum. The highly significant associations between the testis (GDS2098) and ovary (GDS2203) reflect known molecular similarities between male and female gonad development, especially before gestation day 10.5 [28,29]. Profiles comparing later stages in development are not linked between the sexes (e.g., starred profile in Figure 4B).

### Application to Nanog knockdown in embryonic stem cells

**Figure 5:**
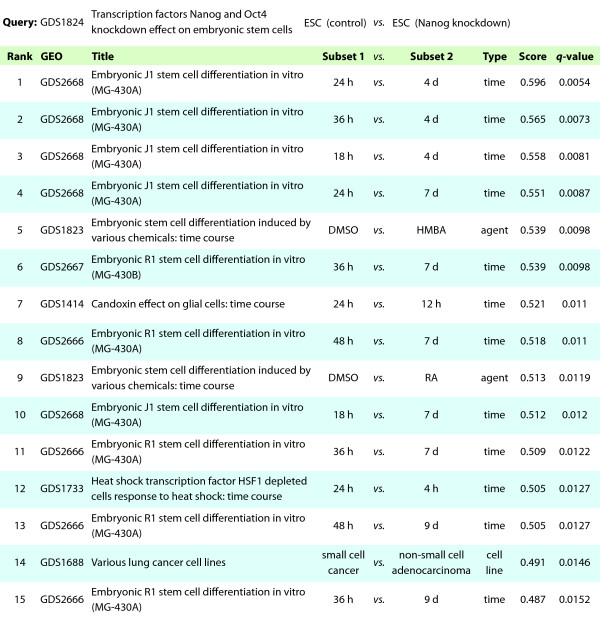
**Search results for Nanog knockdown**.

This search method allowed us to simultaneously investigate a wide range of perturbations, conditions, and comparisons using the hypothesis that experiments that differentially express similar genes and pathways would also share functional phenotypic relationships. To assess the utility of this approach, we used data from GDS1824 to investigate the effects of Nanog knockdown in embryonic stem cells (ESCs) [30]. We created a DE profile comparing Nanog knockdown to control in mouse ESCs, and queried all GEO DataSets to identify other experiments that have similar differential expression patterns. Because the transcription factor Nanog is required for the maintenance of pluripotency in ESCs [31,32], we hypothesized that this search would find profiles comparing embryonic stem cells to differentiated cells. Indeed, ten of the top fifteen matching profiles consisted of experiments comparing less differentiated to more differentiated mouse embryoid bodies of various genetic lineages (Figure 5). In matching experiments, differentiation was induced by removal of LIF (leukemia inhibitory factor) [33], a cytokine necessary to maintain the undifferentiated state of ESCs [34]. The Nanog knockdown search also identified comparisons from GDS1823, also from Loh et al. [30], where ESC differentiation was induced by drug treatment with retinoic acid (RA) or hexa-methylene-bis-acetamide (HMBA).

In addition to mouse ESC datasets, this search produced interesting comparisons with different experimental systems. Result 14 supports a similarity between Nanog knockdown and the comparison of non-small cell lung carcinoma (NSCLC) to small cell lung cancer (SCLC). SCLC, the more aggressive disease, has been linked with expression of stem cell factor [35] and the Hedgehog signaling pathway [36]. These relationships suggest that, in a broad sense, SCLC compared to NSCLC may have a more stem-like transcriptional program.

**Figure 6:**
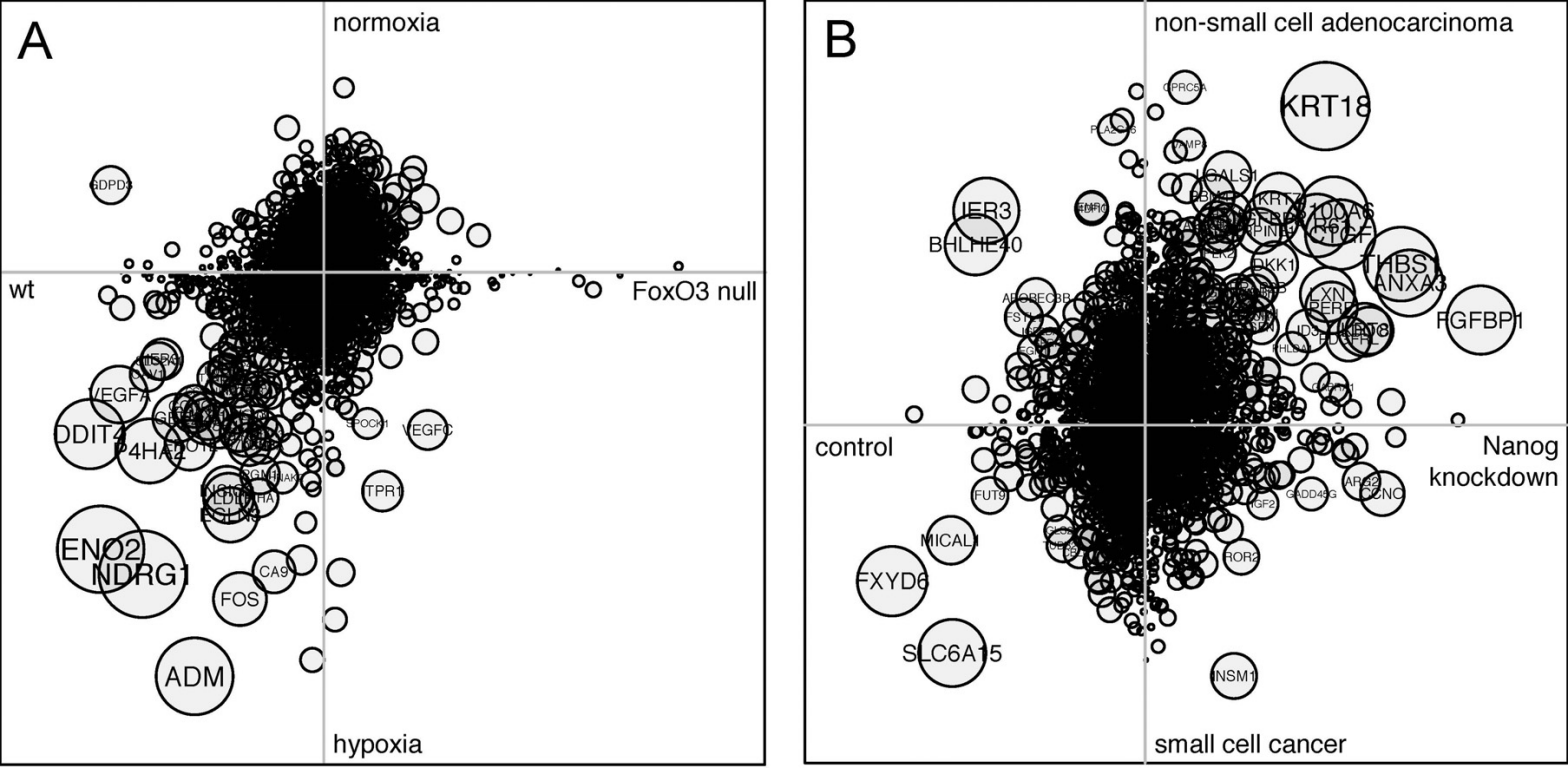
**Gene-level analysis of transcription factor search results**. Scatterplots comparing the log fold-change of each gene shared by two differential expression profiles. Expression values are centered and scaled. The area of each circle is proportional to the contribution that the gene makes to the final correlation score, and thus is a function of the magnitude as well as the significance of differential expression. (A) Comparison of GSE18326: FoxO3 null versus wild type and GDS2758: normoxia versus hypoxia. (B) Comparison of GDS1824: Nanog knockdown versus control and GDS1688: non-small cell adenocarcinoma versus small cell cancer.

Our method also identifies the genes that drive the correlation between two profiles. These genes have the most significant coordinated changes in the two experiments. When we examined the genes driving the correlation for Result 14, we found cytokeratin KRT18 overexpressed in both Nanog knockdown compared to control and in NSCLC compared to SCLC (Figure 6B), fitting previous examinations of KRT18 by immunohistochemistry [37,38]. On the other end of the spectrum, we found the relatively uncharacterized gene FXYD6, a regulator of N, K-ATPase [39]. FXYD6 is down-regulated during Nanog knockdown (see Additional file 5) and up-regulated in several SCLC cell lines (see Additional file 6). This suggests that FXYD6 plays a role in the transcriptional programs in common between embryonic stem cells and SCLC.

### Application to FoxO3 knockout in neural stem cells

**Figure 7:**
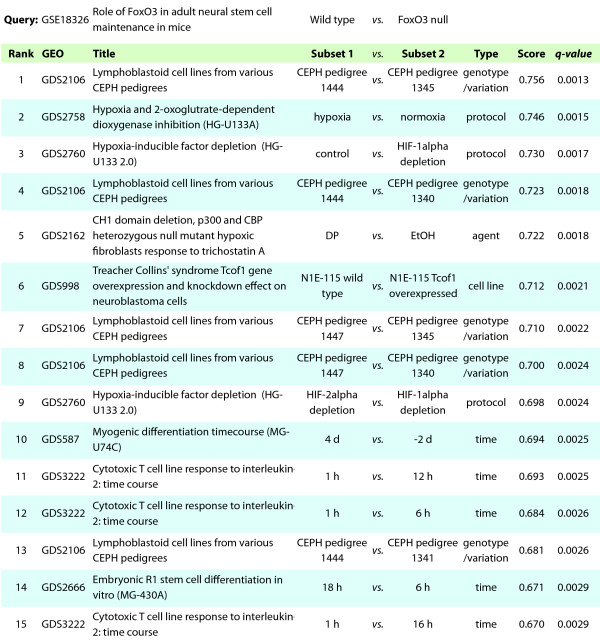
**Search results for FoxO3A knockout**.

To evaluate the predictive potential of our search method in another system, we examined the effects of FoxO3 knockout in neural stem cells (NSCs). FoxO3 regulates NSC homeostasis by preventing premature differentiation and controlling oxygen metabolism [40]. Throughout the body, the FoxO family of transcription factors regulate a wide variety of cellular processes including glucose metabolism, cell cycle arrest, differentiation, and detoxification of reactive oxygen species (ROS) [41,42]. We created a DE profile comparing wild type to *FoxO3^-/- ^*adult mice using normalized data from GSE18326. A query of GEO DE profiles yielded numerous significant results, the most significant of which are shown in Figure 7. Several matching profiles (Results 2, 3, 5 and 9) implicate FoxO3 in hypoxia response: data from GDS2758 and GDS2760 compare MCF-7 breast cancer cells under hypoxic and normoxic conditions as well as with siRNAs targeting hypoxia-inducible factor 1 (HIF-1*α*) and HIF-2*α*. Bakker et al. found that FoxO3 is activated in response to hypoxic stress in mouse embryonic broblasts (MEFs), and furthermore that this activation requires functional HIF-1*α *[43]. Renault et al. also found that FoxO3 is required for the expression of hypoxia-dependent genes in NSCs [40]. GDS2162 (Result 5) compares p300 and CBP null MEFs in response to dipyridyl (DP) or control (EtOH). DP, a hypoxia mimetic, induces HIF-1*α *[44] and thus potentially FoxO3. In all four hypoxia-related profile matches, therefore, the direction of the comparisons accurately predicts known FoxO3 biology. To further probe the relationship between FoxO3 and hypoxia, we examined the genes responsible for the high correlation between our FoxO3 query and Result 2. Predictably, we found genes associated with both hypoxia and FoxO3 signaling (Figure 6A). For instance, DDIT4 and NDRG1, both of which have been found previously to be activated during hypoxia [45,46] also contain FoxO binding motifs in their regulatory regions [40].

Other matches from the FoxO3 search (Results 11, 12, 15) point to a role for FoxO3 in cellular response to cytokine interleukin-2 (IL-2) stimulation. All three of these matching profiles compare cytotoxic T cell line (CTLL-2) at 1 hour after IL-2 stimulation to a later time point (6, 12, or 16 hours). From the direction of these comparisons, we would predict that IL-2 stimulates a transcriptional program that is similar to that of FoxO3 knockout. Indeed, IL-2 signaling leads to phosphorylation and inactivation of FoxO3 in CTLL-2 cells [47], confirming this hypothesis.

## Discussion

In optimizing our data processing and search pipeline, we found that linear combinations of gene expression features derived in a separate compendium benefited our analysis. The most effective dimension reduction technique involved projecting each DE profile into a feature-space identified by independent component analysis. We previously used ICA to identify fundamental components of human gene expression from a large compendium of 10,000 arrays, of which only a small subset overlap with the experiments examined here [27]. The ICA projection method reduced the set of features from on the order of 20,000 to less than 500, allowing for rapid indexing and searching of large libraries of differential expression profiles. Furthermore, this approach outperformed module-based methods, possibly because the linear model incorporated data from all of the genes rather than only those that participate in discrete gene sets. Despite the fact that these ICA features were derived in human data, they proved robust in identifying and ranking experiments in closely related species as well. Thus, our results support previous findings that gene expression features derived in one compendium can be useful for interpreting data from new datasets [48].

To calculate similarities between differential expression profiles, we introduced a novel weighting scheme that incorporates information about a feature's significance of differential expression. This approach provides an intuitive means for emphasizing the contributions of features that are significantly differentially expressed in both experiments, which may represent the most relevant common biology. At the same time, the weighted correlation incorporates even genes that are not significantly differentially expressed, potentially capturing the effects of broader transcriptional changes. We observed that this scheme worked well with Pearson correlation, but did not perform as well when combined with rank-based correlation. Future work will characterize the behavior of this similarity measure on a larger scale.

We used the most successful data processing pipeline to index all GEO DataSets. Our results with transcription factor experiments suggest that this approach can provide predictions for genes, phenotypes and perturbations that share functional similarities with a query experiment. Analysis of Nanog knockdown in ESCs successfully identified other ESC differentiation time courses, induced by a variety of factors, from amongst almost 10,000 other profiles (Figure 5). The same search predicted a link between small lung cell carcinoma and ESC transcriptional programs. For a less well characterized transcription factor, FoxO3, our method also succeeded in recapitulating known biology across species and experimental systems. Although it is clear that FoxO3 has lineage-specific effects [42,49], we identified a role for FoxO3 in hypoxia response that appears to transcend tissue type [40,41]. For uncharacterized comparisons, this information has the potential to provide useful hypotheses for phenotypes and pathways to investigate.

As in more traditional microarray analyses, however, interpretation of the most significant genes identified by our weighting scheme remains difficult. Our analysis of the FoxO3 search revealed a number of genes involved in both hypoxia and FoxO3 signaling, linking these two pathways. However, the top genes in the Nanog knockdown search failed to reveal convincing pathways that might explain the relationship between small lung carcinoma and ESCs. While we focus on the interpretation of several individual genes in this study, future efforts may benefit from the use of gene set enrichment tools to find pathways that are significantly represented in the top gene list.

As experimentalists continue to explore and deposit information about cellular processes and perturbations, the utility of content-based search approaches will increase. With a larger bank of transcriptomic data and a high chance of identifying overlapping and functionally related biology, an "experiment-omic" screen might be the first step in characterizing a novel dataset. To realize this, further ontological indexing of expression databases may also be necessary [50]. Several groups have already begun to integrate expression with textual phenotype data to enable gene function prediction [51] and automatic disease diagnosis [14] from large databases. Even for expression-driven methods, controlled annotations for experimental variables, tissue types, and culture systems would allow for more accurate assessments of functional relevance. Finally, ontological indexing of textual annotations will enable the creation of more sophisticated connectivity maps linking not just diseases and drugs, but also gene knockdowns, over-expression studies, and genotype comparisons. These ontology-informed studies may not only search public repositories based on gene expression, but also provide meta-analysis across phenotypic categories.

## Conclusions

We have explored computational methods needed to search large repositories for relevant experiments based on differential expression, using an experiment as a query. While previous studies use hard thresholding to select gene sets of interest [4,11,13], we propose a data-driven approach that uses information from all shared genes to compare two experiments. Differential expression profiles containing scores for each gene or feature were generated and compared using correlation metrics, following the hypothesis that this direct and intuitive method would perform well across diverse datasets. In a collection of 32 experiments comparing normal to diseased tissue, we achieved an average AUC of 0.737 for retrieving experiments that measure the same disease. We further demonstrated the ability of our method to identify functionally relevant experiments from a large database of studies. Future work will include implementing the principles learned here into a web-based application. Public deployment of these methods will enable discoveries in drug repurposing, disease classification, and systems repositioning as we explore the molecular underpinnings of diverse biological processes and phenotypes.

## Methods

### Disease compendium

From a previous collection of disease-associated NCBI GEO microarray experiments [5], we collected 1,278 processed arrays comprising 32 experiments that compared normal to diseased tissue for Duchenne muscular dystrophy, breast cancer, and Huntington's disease. These experiments represented a variety of species, platforms, tissues, and normalization techniques, factors which might strongly influence the clustering of expression data.

### Differential expression profiles

In transcriptomic studies, differential expression analysis identifies the genes and biological processes that vary between two samples. To represent this information from different datasets in a standardized manner, we mapped probesets to Entrez Gene identifiers using AILUN [52] and generated differential expression (DE) profiles for each comparison using Bioconductor software [53]. Here, a DE profile consists of a list of features (e.g., genes) each with an associated score (e.g., fold change). For each comparison, we represented this differential expression score in three ways.

#### Log fold-change profile

We converted all microarray data to log values by examining the maximum and minimum values of the normalized probe-level data and applying log_2 _transformation as needed. We aggregated probes to genes using the fixed effects meta-estimate, calculating an average for each gene weighted by the variance of each probe [54]. We calculated the fold-change difference between normal and disease by averaging samples within each group.

#### *P*-value profile

Probes were aggregated as for the log fold-change method. For each gene in each experiment, we determined the probability that the gene was differentially expressed with an empirical Bayes moderated *t*-statistic implemented in the *limma *R package (version 2.16.5) [55]. We corrected for multiple hypothesis testing using the Benjamini-Hochberg method [56]. For DE profiles represented in terms of a reduced set of features (see below), we applied *limma *to assess the differential expression of that feature.

#### Rank profile

For each sample in each experiment, we ranked probes based on their raw expression score, then averaged all scores for a probe to create a single score for normal and disease sample groups. We mapped from probes to genes by finding the median of the subtractive difference between all pairwise combinations of probes for the same gene in normal and disease.

### Dimension reduction

For all three DE profile representations, we mapped genes to their human homologs using NCBI Homologene, removing genes that did not have one-to-one homologs between species (Additional file 7). While removing species-specific genes may result in loss of important biological information, we hypothesized that comparing global, conserved patterns of gene expression between experiments would prove sufficient to predict functional associations (see Additional file 1 for data on the number of genes mapped for each dataset discussed in the manuscript). Next, we applied one of two methods of dimension reduction.

#### Projection onto independent components

We previously used independent component analysis (ICA) to identify fundamental features in human gene expression space by analyzing a compendium of 9,460 heterogeneous human microarrays run on the Affymetrix HG-U133 Plus 2.0 platform [27]. Briefly, we applied hierarchical clustering to our compendium to normalize the contributions of over- or under-represented conditions, applied independent component analysis to the normalized data, and aggregated the results over 20 runs using the partitioning around medoids clustering algorithm [57]. The resulting 423 components provide a data-driven feature space on which to map new gene expression data. For each DE profile, we considered the common genes between the experiment and the gene-to-component mapping, then projected the DE profile into ICA feature space using:

(1) $$A=S^{T}X,$$

where *A *is the final reduced profile (423 features), *S *is the component matrix (components × genes), and *X *is the original profile in gene-space.

#### Fixed effect meta-estimate

To evaluate the performance of the ICA projection method, we also used a set of known features to reduce the dimensionality of our DE profiles. Given a collection of gene sets, we calculated a meta-score for each gene set using the fixed-effect meta-estimate, which represents an average across all genes in the set weighted by their inverse variance [58]. This method summarizes the contributions of functionally coherent gene sets, and may be appropriate for expression analysis. We used MSigDB v2.5, a well described collection of 5,452 gene sets most often used in conjunction with GSEA [16]. For comparison, we also derived gene sets from the ICA features described above: for each independent component, we created a module from all genes that scored three standard deviations above the mean in one direction. These 423 modules represented data-derived functionally coherent gene sets as determined by GO enrichment [27].

### Similarity measures

We compared DE profiles in gene- or feature-space using similarity measures based on Pearson correlation coefficient and Spearman rank correlation coefficient. We used three weighting schemes. First, unweighted correlations are typically used in microarray clustering and search applications. However, this approach does not incorporate information about the variability of a gene, either across the compendium or within each dataset. Thus we tested a weighting scheme that accounts for the magnitude as well as the variance of a gene's change. We reasoned that genes with high variability across the compendium should be weighted lower than genes with low variability; that is, a change of the same magnitude should be more significant for a gene with low variance than for a gene with high variance, since its relative deviation from the mean would be higher. To account for this, we calculated an inverse-variance weighted correlation, where each feature is weighted by the inverse of its variance across the entire compendium. Finally, we explored the possibility of weighting each gene by a function of its differential expression in the two datasets. Intuitively, a gene that is differentially expressed in both datasets should receive more weight than a gene that is differentially expressed in one or neither dataset. While Pearson correlation already rewards high magnitude changes, we chose to further weight genes by their *p*-value of differential expression to incorporate the inter-dataset variance as well as the magnitude. We calculated the weights for this *p*-value weighted correlation using:

(2) $$w_{i}={\left[-log\left(p_{i1}p_{i2}\right)\right]}^{1/C},$$

where *w_i _*is the weight for feature *i*, *p_ij _*is the FDR-corrected empirical Bayes *p*-value for experiment *j*, and *C *is a scaling factor. For this work, we empirically chose *C *= 2 because it delivered the best clustering of our disease compendium (data not shown). We used the ROCR package [59] to evaluate the performance of various data processing methods.

### GEO DataSet search

To search GEO for experiments with similar transcriptional patterns, we indexed all GEO DataSets (GDSs). We downloaded processed data from GEO and used the GDS "Value type" field to transform the data to log_2 _space. Each GDS is manually annotated with one or more factors, e.g., "disease state" or "time", which outline the experimental conditions that vary between groups of samples. Within each GDS, we compared all combinations of groups for a single factor. For each of these comparisons, we created two DE profiles: one in gene-space, and one in the ICA feature-space described above. We calculated *p*-values for each gene and ICA feature using the empirical Bayes modified *t*-test as described [27]. To search these DE profiles, we used the absolute value of the *p*-weighted Pearson correlation metric, since the direction of the comparison is arbitrary. To assess the significance of DE profile comparisons, we selected 10,000 random pairs of comparisons to serve as a background distribution of correlation scores. We estimated the false discovery rate (FDR) of our search results by calculating the percentage of these random comparisons that exceed a given similarity score. Because this random sampling may include true positive comparisons (e.g., two profiles from the same dataset), our corrected *p*-values may underestimate the significance of new comparisons.

## Authors' contributions

JE, AB and RA conceived of the study. JE and AM designed the experiments. JD provided curated disease datasets. JE performed the experiments and wrote the manuscript. RT conducted preliminary experiments and implemented a prototype web-site. RC contributed code for generating GEO comparisons. All authors reviewed and approved the final manuscript.

## Supplementary Material

Additional file 1 **Evaluation of data representation methods**. We explored three alternative methods for representing differential expression data: log fold-change, normalized rank difference, and adjusted *p*-value significance. Using our disease compendium, we performed leave-one-out cross-validation by using each of 32 experiments to query the others. We generated ROC curves with different combinations of data representation and correlation metrics. Bars and AUC estimates indicate standard errors for curves averaged over all cross-validation trials. Click here for file

Additional file 2 **Precision at 4 for ICA p-weighted Pearson search**. We calculated the "precision at 4" for each experiment in the disease compendium. Red bars show null distribution creating by permuting labels with 95% confidence intervals. Click here for file

Additional file 3 **High resolution network of GEO differential expression profiles**. Vector graphic representation of the network in Figure 4. See Additional file 4 for legend. Click here for file

Additional file 4 **Legend for differential expression profile network**. Legend for Additional file 3. Click here for file

Additional file 5 **FXYD6 Expression in GDS1824**. GEO Gene profile for FXYD6 in GDS1824. See http://www.ncbi.nlm.nih.gov/sites/entrez?db=geo&term=GDS1824[ACCN]+fxyd6. Click here for file

Additional file 6 **FXYD6 Expression in GDS1688**. GEO Gene profile for FXYD6 in GDS1688. See http://www.ncbi.nlm.nih.gov/sites/entrez?db=geo&term=GDS1688[ACCN]+fxyd6. Click here for file

Additional file 7 **Homolog mapping**. Excel Spreadsheet describing the number of genes mapped through Homologene to human for each dataset discussed in the text. Click here for file
