# Supplementary figures and images for: Content-based microarray search using differential expression profiles
Source: BMC Bioinformatics. 2010 Dec 21;11:603. doi: 10.1186/1471-2105-11-603 (PMC3022631; doi:10.1186/1471-2105-11-603)

True Positive Rate (Sensitivity)

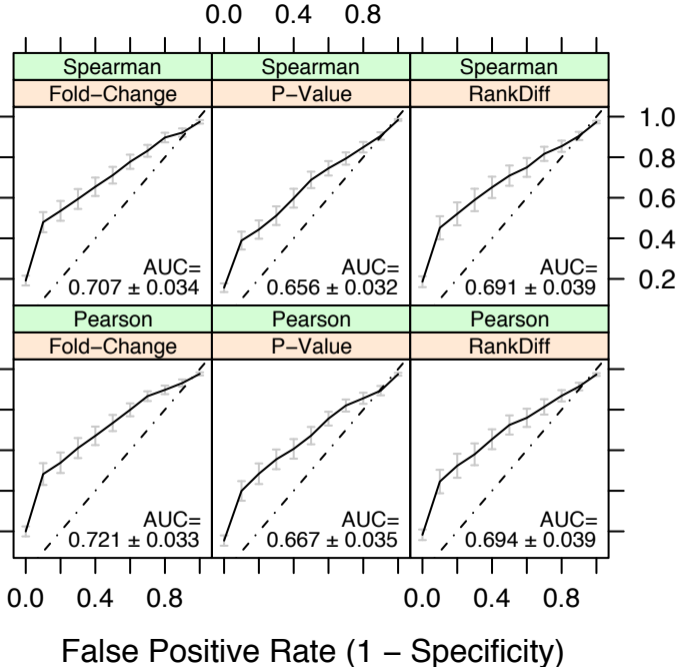

Supplement: Additional file 1 — Evaluation of data representation methods. We explored three alternative methods for representing differential expression data: log fold-change, normalized rank difference, and adjusted p-value significance. Using our disease compendium, we performed leave-one-out cross-validation by using each of 32 experiments to query the others. We generated ROC curves with different combinations of data representation and correlation metrics. Bars and AUC estimates indicate standard errors for curves averaged over all cross-validation trials. [file 1471-2105-11-603-S1.PDF]

Precision at 4 for P-Weighted Pearson Correlation Search, ICA Projection

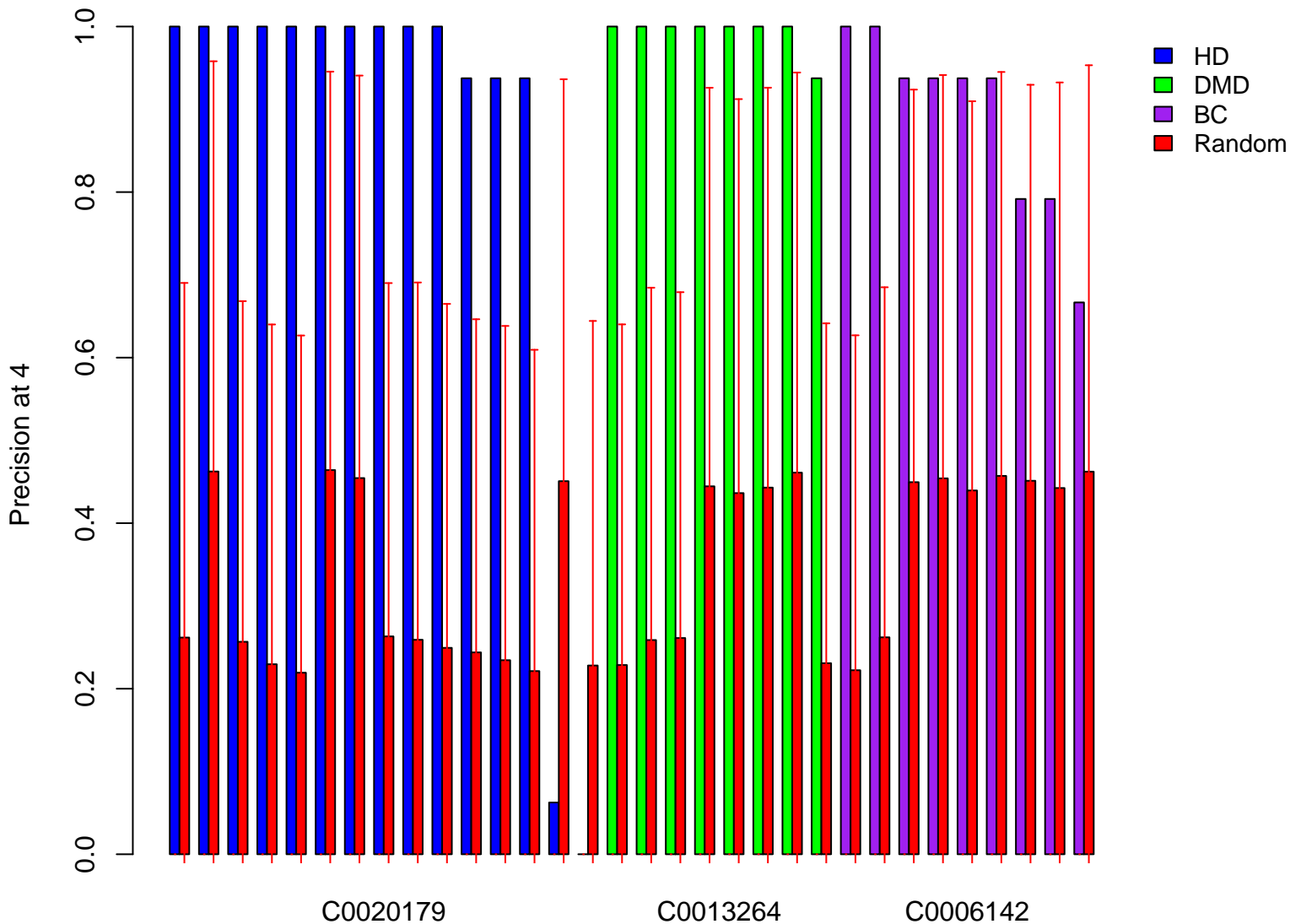

Supplement: Additional file 2 — Precision at 4 for ICA p-weighted Pearson search. We calculated the "precision at 4" for each experiment in the disease compendium. Red bars show null distribution creating by permuting labels with 95% confidence intervals. [file 1471-2105-11-603-S2.PDF]

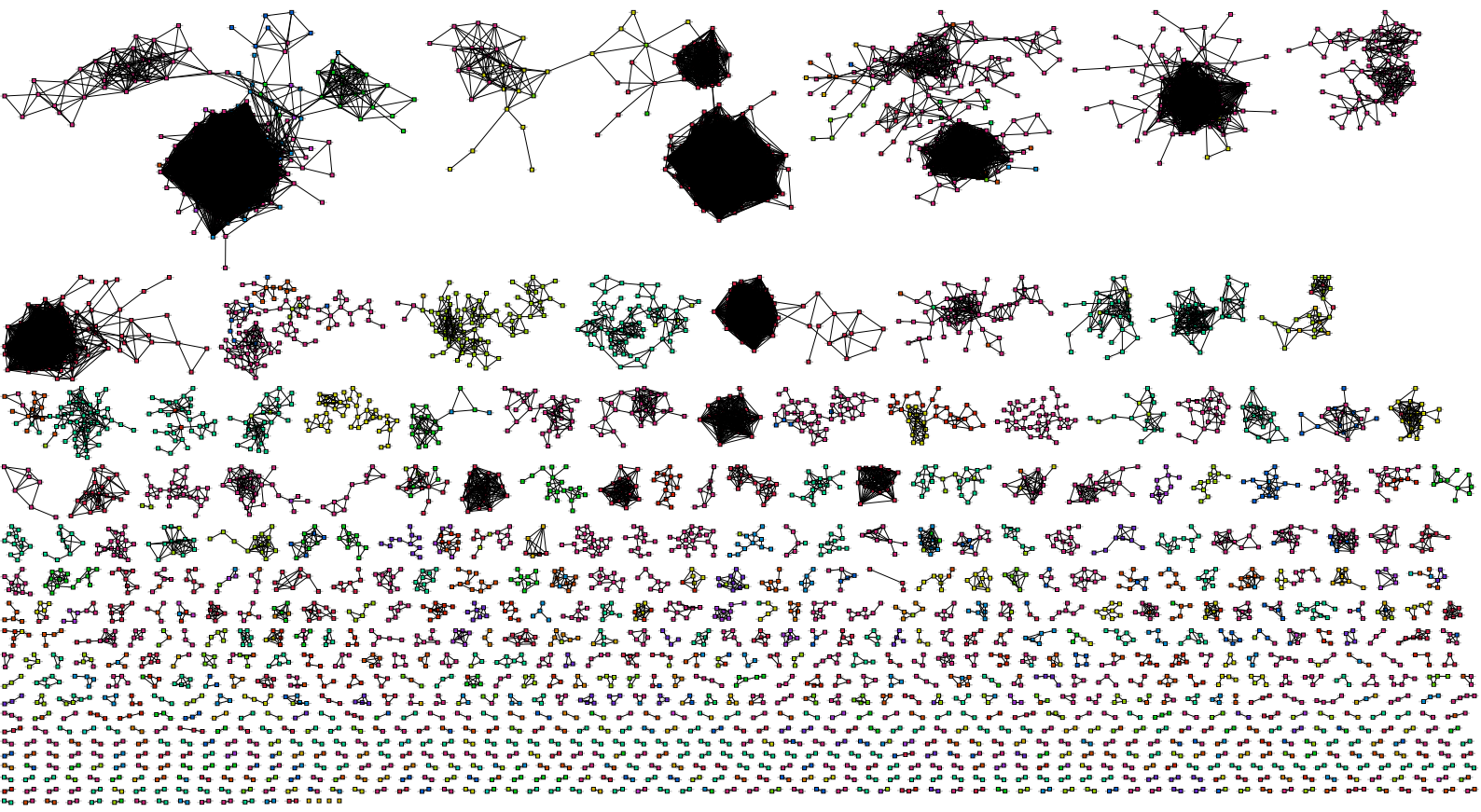

Supplement: Additional file 3 — High resolution network of GEO differential expression profiles. Vector graphic representation of the network in Figure 4. See Additional file 4 for legend. [file 1471-2105-11-603-S3.PDF]

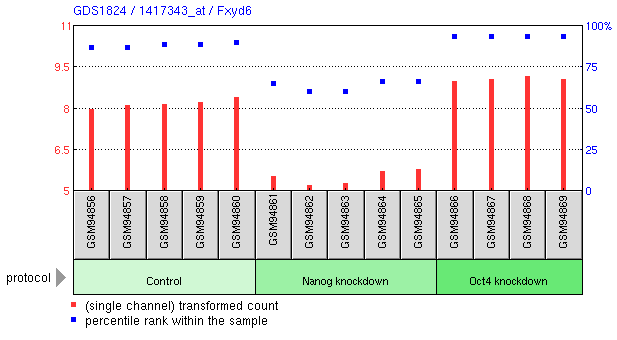

Supplement: Additional file 5 — FXYD6 Expression in GDS1824. GEO Gene profile for FXYD6 in GDS1824. See http://www.ncbi.nlm.nih.gov/sites/entrez?db=geo&term=GDS1824[ACCN]+fxyd6. [file 1471-2105-11-603-S5.PNG]

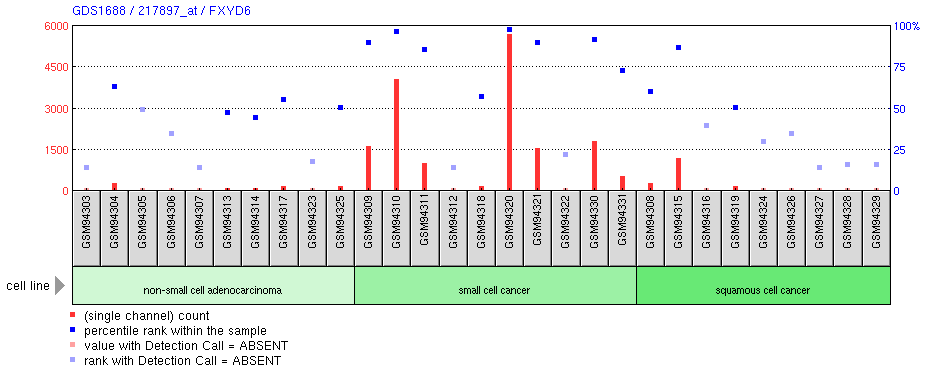

Supplement: Additional file 6 — FXYD6 Expression in GDS1688. GEO Gene profile for FXYD6 in GDS1688. See http://www.ncbi.nlm.nih.gov/sites/entrez?db=geo&term=GDS1688[ACCN]+fxyd6. [file 1471-2105-11-603-S6.PNG]
