# Supplementary material for: Content-based microarray search using differential expression profiles
Source: BMC Bioinformatics. 2010 Dec 21;11:603. doi: 10.1186/1471-2105-11-603 (PMC3022631; doi:10.1186/1471-2105-11-603)
Supplement: Additional file 4 — Legend for differential expression profile network. Legend for Additional file 3. [file 1471-2105-11-603-S4.PDF]

Subset Types

- age
- agent
- cell line
- cell type
- development stage
- disease state
- dose
- gender
- genotype/variation
- growth protocol
- individual
- infection
- isolate
- other
- protocol
- shock
- species
- specimen
- strain
- stress
- temperature
- time
- tissue
